# Visualization and quantification of pancreatic tumor stroma in fresh tissue via ultraviolet surface excitation

**DOI:** 10.1117/1.JBO.26.1.016002

**Published:** 2021-01-09

**Authors:** Phuong Vincent, Petr Bruza, Scott M. Palisoul, Jason R. Gunn, Kimberley S. Samkoe, P. Jack Hoopes, Tayyaba Hasan, Brian W. Pogue

**Affiliations:** a Dartmouth College, Thayer School of Engineering, Hanover, New Hampshire, United States; b Dartmouth-Hitchock Pathology Shared Resource Lab, Lebanon, New Hampshire, United States; c Geisel School of Medicine, Department of Surgery, Hanover, New Hampshire, United States; d Harvard Medical School, Wellman Center for Photomedicine, Boston, Massachusetts, United States

**Keywords:** collagen imaging, microscopy with ultraviolet surface excitation, pancreatic adenocarcinoma, fluorescence imaging, ultraviolet light, photodynamic therapy

## Abstract

**Significance:** The study has confirmed the feasibility of using ultraviolet (UV) excitation to visualize and quantify desmoplasia in fresh tumor tissue of pancreatic adenocarcinoma (PDAC) in an orthotopic xenograft mouse model, which provides a useful imaging platform to evaluate acute therapeutic responses.

**Aim:** Stromal network of collagen prominent in PDAC tumors is examined by imaging fresh tissue samples stained with histological dyes. Fluorescence signals are color-transferred to mimic Masson’s trichrome staining.

**Approach:** Murine tumor samples were stained with Hoechst, eosin, and rhodamine B and excited at 275-nm. Fluorescence signals in the visible spectrum were captured by a CMOS color camera with high contrast and resolution at whole-tumor slice field of view.

**Results:** Fluorescence imaging using UV excitation is capable of visualizing collagen deposition in PDAC tumors. Both fluorescence and histology data showed collagen content of up to 30%. The collagen modulation effect due to photodynamic priming treatment was observed showing 13% of collagen reduction. Necrosis area is visible and perfusion imaging using Texas Red dextran is feasible.

**Conclusions:** The study demonstrates collagen visualization in fresh PDAC tumor samples using UV excitation. This imaging platform also provides quantitative stromal information from fiber analysis and visibility of necrosis and perfusion, suitable for therapeutic response assessment of photodynamic therapy.

## 1 Introduction

The microenvironment of pancreatic adenocarcinoma (PDAC) is well recognized as a highly complex cellular-molecular-stromal milieu that hinders therapeutic response.^1^^,^^2^ The hyperdense desmoplastic nature of PDAC has been associated with drug resistant^3^ cancer progression,^4^ prompting a major direction of targeted therapies focusing on stromal depletion.^5^ However, attempts to alleviate the effects of dense stroma have yielded mixed results,^6^^,^^7^ and it may be that systemic molecular therapies may not be the ideal way to deal with the type of desmoplasia in PDAC. While major mechanistic efforts have elucidated the pathobiological relationship of pancreatic stellate cells with other tumor microenvironment components,^8^^,^^9^ recent findings have called for more attention toward the spatial orientation of particular biomarkers such as immune cells^10^ and fibroblasts.^11^ The study of these components and contributors to the desmoplasia is challenging to examine because of how dynamic the microenvironment is and how hard it is to examine molecular signals and morphology in fresh tissues. In this study, a methodology to image and quantify stroma and some molecular signals in fresh PDAC is examined.

Existing approaches to retrieve quantitative stromal information includes traditional histology, immunofluorescence staining,^12^ and optical imaging methods, such as second harmonic^13^ or birefringence imaging.^14^ These techniques are subject to extensive sample preparation and/or limited specimen size, which inhibits the capability of capturing whole tissue heterogeneity. Fortunately, advancements in fresh tissue optical imaging have shown how it is possible to image intact whole specimens while mapping at the microscopic level. Microscopy with ultraviolet surface excitation (MUSE) developed by Fereidouni et al.^15^ exploits ultraviolet (UV) excitation at wavelengths shorter than 300 nm to provide image contrast when imaging bulk tissues with just thin slice excitation at the surface. In MUSE, exogenous stains excited at 280-nm illumination are applied to highlight different tissue components and to overcome intrinsic autofluorescence, all of which utilize a very simple and cost-effective optical design. While it was reported that stromal components have not yet been well-studied using this imaging technique,^15^ its potential capability of imaging stroma directly from fresh tissue provides an ideal tool for studying the tumor microenvironment heterogeneity and changes to this from response to targeted therapies.

Therefore, this study developed the capability to use UV-fluorescence (UV-fluor) imaging in stromal imaging from fresh tissue of PDAC tumors, which yields strong collagen signals. The work examined BxPC-3, a human-derived tumor cell line orthotopically implanted in xenograft mouse models for analysis of the stromal network of PDAC. Previous studies have confirmed the micro-heterogeneity of collagen in this tumor type and that the collagen density is strongly correlated to the tumor biomechanical stiffness and inversely with vascular perfusion.^16^^,^^17^ The current study examined if equivalent collagen information could also be visualized and quantified directly from fresh tissue imaging as compared to traditional pathology stained fixed tissues. Furthermore, assessment of viable tumor cells and necrotic areas was examined in the same setting from UV-fluor signal of Hoechst staining. If successful, this technique could provide a fast assay platform for *in situ* investigation of therapeutic effects from interventional targeted therapies in a way that does not require pathology processing and post imaging registration. One of our primary goals was to use this tool as an assay of PDAC reaction to photodynamic therapy (PDT)^18^^,^^19^ to examine the subtle changes that can occur with sub-lethal PDT, sometimes referred to as “photodynamic priming” for adjuvant therapies.^20^

## 2 Materials and Methods

### 2.1 Infinity-Corrected Imaging Setup

**Fig. 1:**
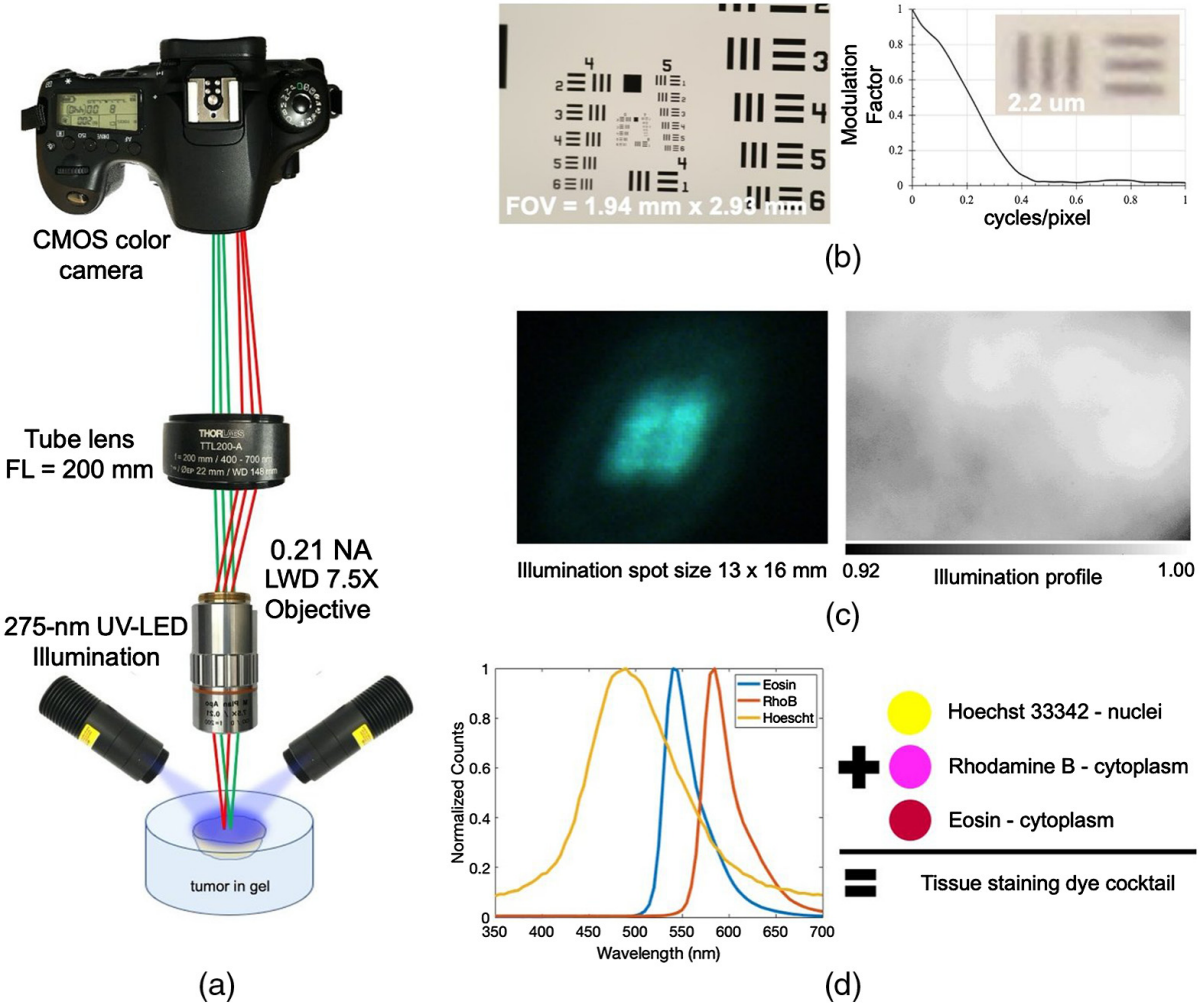
UV-fluor imaging system setup. (a) A schematic of the imaging system with camera tube lens and either 7.5× or 10× objective lens. Illumination sources were two UV-LEDs mounted symmetrically. (b) The imaging field of view and spatial resolution test are shown. (c) The illumination spot size generated by the UV-LEDs is shown with irradiance $10\;{\mathrm{mW}/\mathrm{mm}}^{2}$. (d) Emission spectra of UV-excited staining dyes when illuminated at 275-nm covered the detection range of the RGB camera.

The system utilized the simplicity of infinity-corrected optics and the versatility of a commercial RGB camera [Fig. 1(a)]. Fluorescence signals were focused by a long working distance objective (Mitutoyo, Kawasaki, Japan) onto a 200-mm tube lens (#TTL-200A, Thorlabs), which were captured by a commercial Electro-Optical System (EOS) color camera (EOS 60D, Canon, Japan). Camera settings were initialized by the native EOS Utility, including exposure time, ISO levels, and image output format. The objective was able to fill up the Advanced Photo System type-C sensor size of the camera, given a field of view of ∼1.94×2.93  mm. The imaging system was able to resolve Group 7 Element 6 on the U.S. Air Force Office of Scientific Research 1951 resolution target. The modulation transfer function (MTF) was computed, and resolution was found to be 0.5  μm at 10% contrast [Fig. 1(b)]. Raw image data were obtained by LabVIEW software readout and control.

### 2.2 Ultraviolet Illumination Source

Open-faced, dark-field illumination was exploited using two 275-nm UV-LEDs (#M275L4, Thorlabs) mounted symmetrically to provide a more uniform light distribution. The LED emission was collimated by a 20-mm fused silica ball lens to provide an irradiance of $10\;{\mathrm{mW}/\mathrm{mm}}^{2}$, covering an area of ∼13×16  mm [Fig. 1(c)]. The LED drivers (#LEDD1B, Thorlabs) provided a current of 700 mA to each LED and a trigger signal for hardware synchronization. The LEDs functioned in trigger mode to minimize photobleaching and ensure temperature control. The short 50-μs LED rise time as compared to 50-ms exposure time in addition to a couple of seconds of moving the translational table to a new spatial location provided adequate off time for the LEDs to avoid heading and any significant spectral changes.

### 2.3 Animal Models and Tumor Sample Preparation

All animal procedures were conducted under the protocol approved by the Dartmouth Institutional Animal Care and Use Committee. A total of five athymic nude mice between the age of 6 to 8 weeks were used in this study. They were injected with human tumor cell line BxPC-3 (ATCC, Cat# CRL-1687). The pancreas was exposed, and tumor cells were injected with a 1∶1 ratio of Matrigel. BxPC-3 required 5 to 7 weeks for tumors to reach ideal imaging size of 1 cm in diameter. The mice were on purified diet to reduce autofluorescence from chlorophyll-based food consumption. After the tumors reached imaging size, the mice were anesthetized and sacrificed. Dextran Texas Red (TR) (Thermo Fisher Scientific, Cat# D1864) was intravenously injected 1-h before sacrifice to demonstrate the capability of perfusion imaging using the same imaging setup. Tumors were resected, embedded in 2.5% agar, and sliced in half. A layer of phosphate-buffered saline (PBS) was applied on the tumor surface to maintain proper hydration.

### 2.4 Photodynamic Treatment

Two out of five mice were injected with 0.5-mg/kg Visudyne photosensitizer (1-h before PDT treatment with 690-nm laser at a dose of $75\;{J/\mathrm{cm}}^{2}$ and irradiance of $100\;{\mathrm{mW}/\mathrm{cm}}^{2}$. The light was given by a fiber optic cable at the exposed tumor site. The mice were sacrificed two days after the treatment.

### 2.5 Tumor Staining

Conventional pathological fluorophores such as Hoechst 33342, eosin, and rhodamine B (Sigma) were used as exogeneous stains on fresh and fixed tissue samples. Theses fluorophores could be excited by a deep UV illumination to highlight tissue morphology [Fig. 1(d)]. In this study, a combination of Hoechst 33342 (0.5  mg/ml in PBS), eosin (1  mg/ml in PBS), and rhodamine (0.2  mg/ml in PBS) was adequate for PDAC stromal imaging. The tissue sample was submerged in this dye combination for 30 s then washed off by PBS for 1-min.

### 2.6 Image Acquisition, Depth of Field Correction, and Stitching

**Fig. 2:**
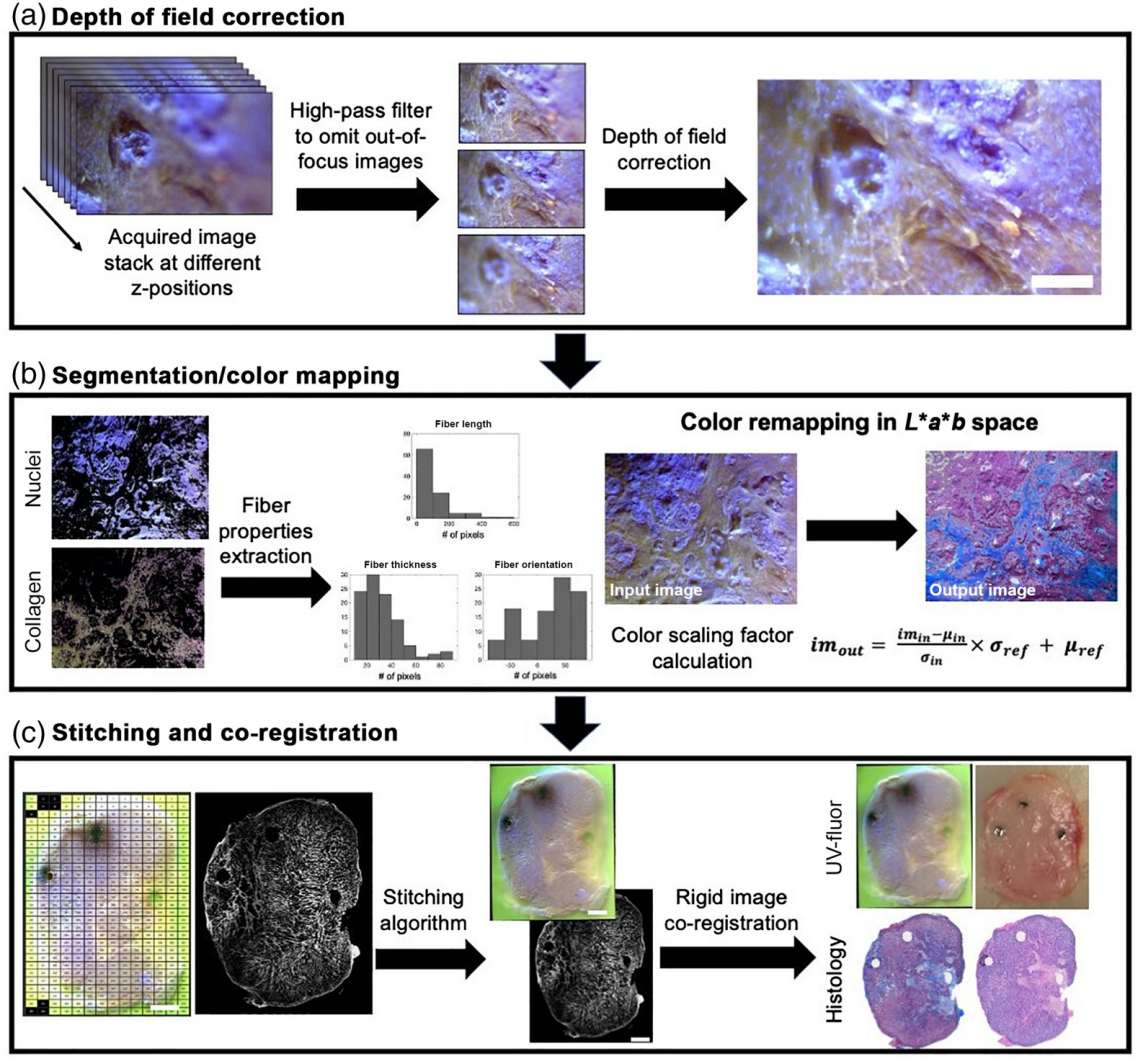
Schematic of primary image processing steps. (a) At each location, an image stack was acquired by moving tissue samples on a motorized translation stage. High-pass filtering was applied to reduce out of focus information. DOF correction was then applied. Scale bar=100  μm. (b) Nuclei and collagen were identified by color segmentation in HSV space. Fiber analysis was done on segmented collagen to yield information such as fiber length, thickness, orientation, and crosslinking profile. UV-fluor images were also color-map transferred to mimic the color palette of MT staining (blue collagen, dark purple nuclei, and light pink cytoplasm). The color remapping process was executed in L*a*b space, utilizing the mean and standard deviation of each channel to create color a scaling factor. (c) Image stitching was performed with 10% overlap to create a whole-tumor view. Rigid image co-registration was performed on the whole-tumor size UV-fluor images, histology images, and brightfield photos of freshly resected tumors to facilitate comparison across imaging modalities. Scale bar=2  mm.

Image acquisition was automated by LabVIEW to allow for synchronization of illumination sources, camera shutter, and a motorized three-dimensional (3D) stage. At each xy coordinate, a series of images taken at seven 6.3-μm vertical increments provided an image stack for depth of field (DOF) correction [Fig. 2(a)]. While 6.3-μm step size was the limit of our current vertical stage, smaller vertical step-size and more acquisitions for each location would result in higher image quality. Due to the high stiffness nature of PDAC tumors, tissue surface irregularity became a significant issue. Therefore, optical sectioning at multiple z-locations was necessary to maximize the number of in-focus pixels. Then, the DOF correction algorithm written by Aguet et al.^21^ was executed in MATLAB. Image stitching was implemented by Microsoft Image Composite Editor Software with 10% overlap to produce a whole tissue sample field of view [Fig. 2(c)]. After imaging, the tumors were formalin fixed and prepared for staining with Masson’s trichrome (MT) to visualize collagen and Hematoxylin and Eosin (H&E) to verify necrosis areas, which was essential for tumors that were treated with PDT to evaluate treatment effects. Both the UV-fluor data and the MT data acquired for collagen analysis were imaged with a 10× magnification lens.

### 2.7 Tumor Parameter Identification

#### 2.7.1 Structural segmentation

Color segmentation in hue-saturation-value (HSV) space was used to distinguish nuclei and collagen [Fig. 2(b)]. Image data were histogram stretched, then segmented in MATLAB. Hue (h), saturation (s), and value (v) cutoffs for purple nuclei were (0.449<h<0.770, 0<s<1, and 0.656<v<1) and for green collagen (h>0.750 or h<0.464, 0<s<1, 0.341<v<1). Vacuoles that appear as round black holes in UV-fluor data were also segmented (h>0.773 or h<0.113, 0.475<s<1, and 0<v<0.477). The distinctive color cutoffs produced no overlays between structures. Any segmented regions that contained fewer than 200 pixels were considered part of the cytoplasm since their sizes were considerably smaller than a nucleus, a collagen fiber, or a vacuole. These criteria for classifying structures in the HSV space were exclusively selected for pancreatic tumor samples, thus further modifications should be considered for other tissue structures.

#### 2.7.2 Collagen analysis

**Fig 3:**
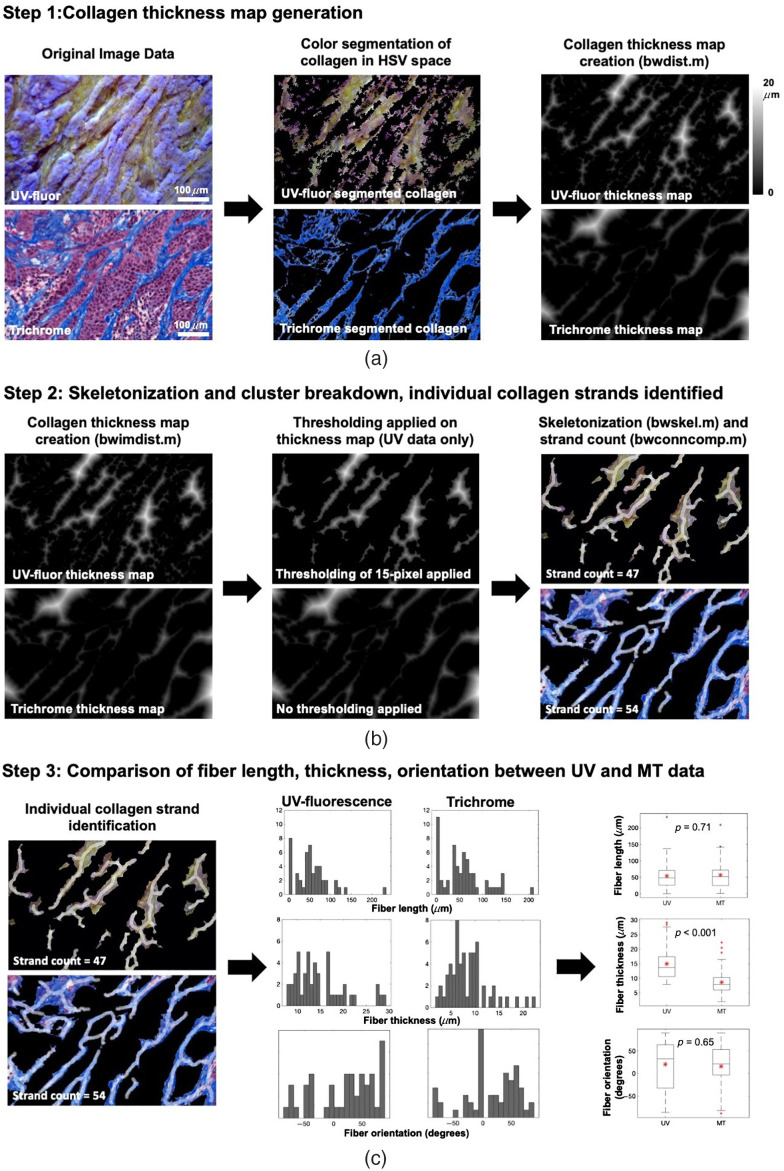
Demonstration of collagen segmentation and quantification methods for UV-fluor data with validation testing on trichrome data, summarized in three main steps. (a) Step 1 generated collagen thickness maps using the MATLAB function bwdist.m performed on segmented collagen in HSV space. (b) Step 2 resulted in skeletonized maps of collagen in combination with thickness maps. Necessary refinements on UV-fluor data were highlighted. A thresholding value was applied on the UV-fluor collagen thickness map to eliminate inaccurate connections of clusters and small cross-sections of collagen due to tissue cut orientation. Skeletonization using bwskel.m and cluster breakdown using bwconncomp.m yielded individual collagen strands. (c) Fiber analysis was performed on each collagen strand to measure the length, thickness, and orientation. Statistical analysis to compare fiber analysis between UV-fluor and trichrome data was conducted to validate the feasibility of using UV-fluor imaging data to obtain quantified collagen information.

Collagen analysis was performed in MATLAB. The binary mask of segmented collagen in the previous step was refined prior to collagen analysis. In cases in which nuclei were positioned on top of a collagen, their superficial position severely interfered with skeletonization and crosslinking analysis, therefore, they needed to be removed initially using function bwareaopen.m to perform hole removal on the collagen binary mask. After that, collagen thickness map was computed using the function bwdist.m, which calculates the distance of all positive pixels to their nearest background pixel [Fig. 3(a)]. Thresholding was applied to the UV-fluor collagen thickness map for two purposes: (1) to eliminate small collagen areas that were the cross-sections of long strands due to the orientation of tissue cuts and (2) to reduce the blur effect due to out of focus pixels that could inaccurately clump the nearby strands together. After these refinements, skeletonization was performed on the collagen thickness map using bwskel.m. Intersections of collagen strands were identified using branchpoints operation with bwmorph.m, and these intersection points were used to break down the collagen network into separate individual strands [Fig. 3(b)]. For each collagen strand, average fiber thickness was determined by twice the average distances of all pixels of the skeletonized centerline to their nearest background pixel. Calling regionprops.m with relevant measurement properties provided analysis for fiber count, length, and orientation [Fig. 3(c)]. The whole algorithm was applied to MT data as validation testing to ensure accurate fiber analysis on UV-fluor data. Manual segmentation and quantification of fibers were performed on a panel of 10 test images for both UV-fluor and MT image sets, which were chosen to cover a wide range of collagen fiber lengths, thicknesses, and orientations. After the algorithm was validated, auto-segmentation was performed with manual checking of key intermediate results such as the collagen thickness maps and skeletonized maps, for all regions of interest (ROIs) reported in Sec. 3.

### 2.8 Color Remapping

After structural segmentation described in the previous step, color remapping was implemented to adjust from UV-fluor color palette to a more conventional color scheme of MT staining. The color transfer process described in Reinhard et al.^22^ was executed in MATLAB. Image data were converted to L*a*b space (*L* for lightness, a for red to green color values, and b for yellow to blue color values). For each structure (collagen, nuclei, or cytoplasm), the mean and the standard deviation of each color channel were computed to create a scaling factor between the UV-fluor and the MT data sets [Fig. 2(b)]. Output images resembled the color scheme from MT staining.

### 2.9 Statistical Analysis

A two-sample student’s t-test was employed in MATLAB without the assumption of equal variances between the tested samples to determine the difference in means using a two-tailed analysis and α=0.05.

## 3 Results

### 3.1 Stromal Content of Fresh PDAC Tumors can be Visualized Microscopically and Macroscopically Using UV-fluorescence Imaging

**Fig. 4:**
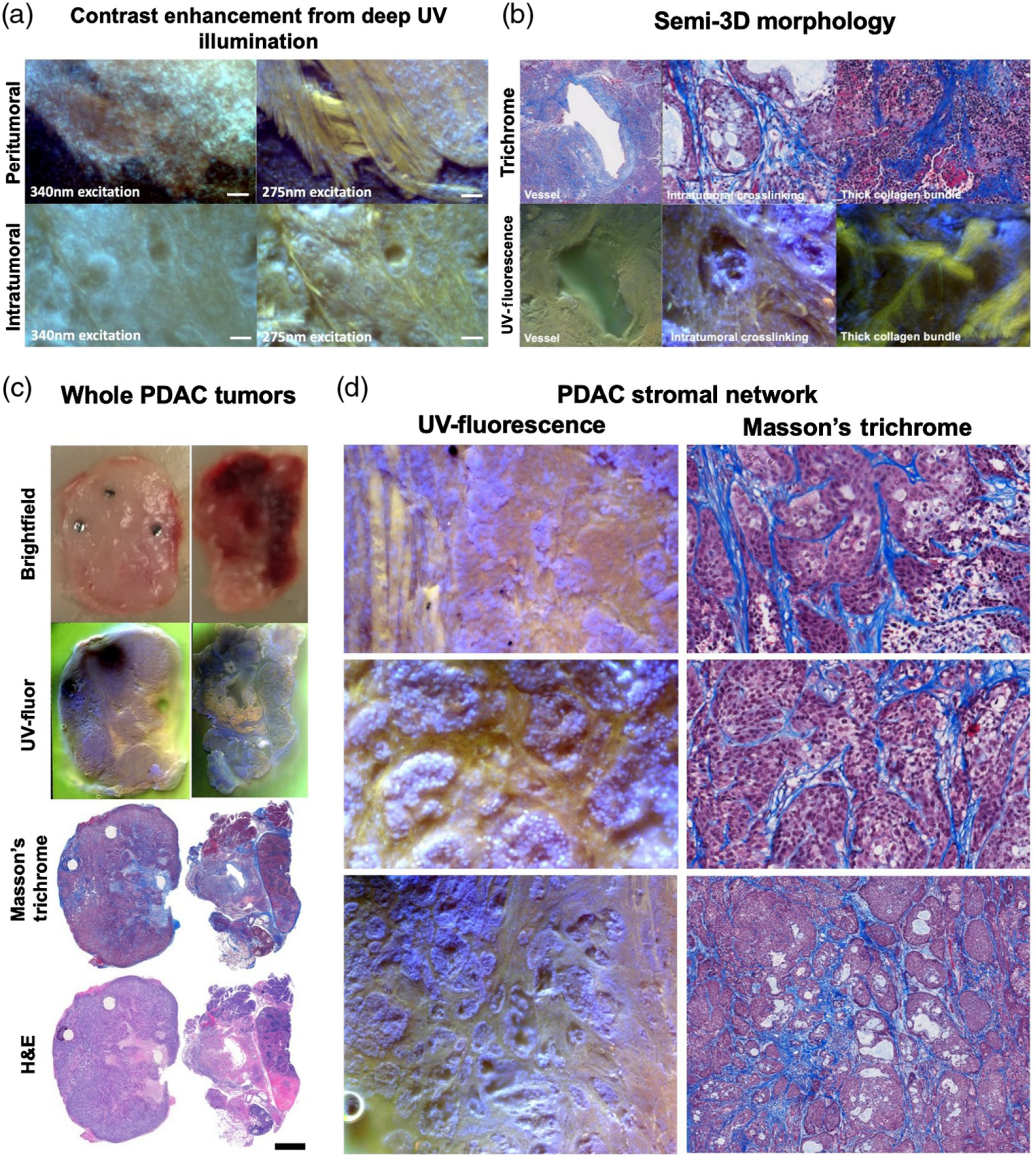
UV-fluor imaging is capable of capturing stromal content in PDAC tumors. (a) Deep UV-illumination below 300 nm provides excellent image contrast, revealing morphology at the microscopic level. Collagen content from peritumoral and intratumoral regions are illustrated, scale bar=100  μm. (b) Optical sectioning and DOF correction provide semi-3D depth information, which highlights 3D structures, such as vessel, collagen crosslinking, and bundling. (c) Rapid imaging allows tumor visualization at macroscopic level, scale bar=2  mm. (d) Demonstration of strong yellow-green collagen signal was obtained from an RGB camera of fresh tissue imaging, and this is compared to MT staining data.

Figure 4(a) shows the contrast enhancement of illuminating samples below 300 nm. For the same tissue specimen, 275-nm excitation reveals morphological features that are undetectable at 340-nm excitation. Collagen strands in yellow-green color at both peritumoral and intratumoral regions are observable, suggesting that UV-fluor imaging is capable of detecting thin collagen strands with diameters down to 5  μm. Since image acquisition required optical sectioning of the specimens at different depths, the data after being DOF corrected also yielded semi-3D morphology [Fig. 4(b)]. Structural components such as blood vessels, collagen crosslinking, and collagen bundling can also be visualized with this imaging technique. Due to rapid imaging, a translation stage coupled with the imaging system could produce whole-specimen images with microscopic resolution. The stitching algorithm provides macroscopic image data, from which regions of necrosis could be easily identified as observed in Fig. 4(c). The light yellow/orange regions in the UV-fluor images are well-aligned with regions of necrosis inferred from H&E staining. A closed-up look at the images revealed collagen content in the tumors, as shown in Fig. 4(d). Side-by-side comparison of UV-fluor data and corresponding MT staining from the same specimens shows great agreement of where tumor collagen is located and the complexity of collagen network.

### 3.2 UV-fluorescence Image Data with a 3-Dye Staining Technique Can Mimic Masson’s Trichrome Staining

**Fig. 5:**
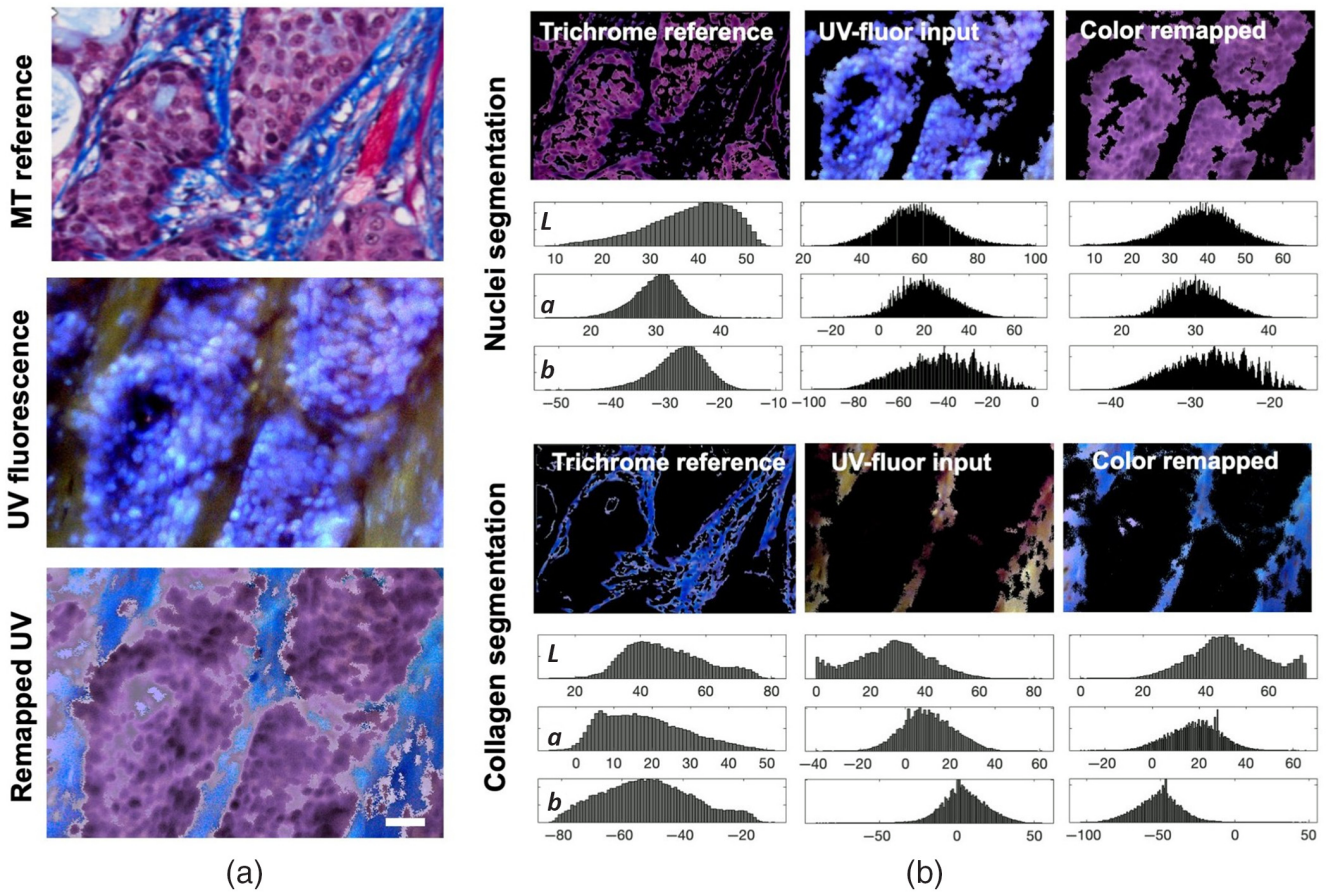
Color remapping of UV-fluor images allows visualization using MT staining color scheme. (a) UV fluorescence image after color remapping showed collagen in blue and nuclei in dark purple, similar to MT staining results, scale bar=30  μm. (b) Illustration of color remapping in L*a*b color space, based on structural segmentation. Corresponding histograms of reference, input, and output images show that the distribution of color intensities and hues was preserved.

Image data acquired by UV-fluor imaging shows that collagen network and viable tumor cells are distinct from each other, due to different emission peaks of Hoechst 33342 and eosin. Figure 5(a) highlights the feasibility of converting UV-fluor data into MT equivalent color scheme, i.e., collagen as blue, nuclei as dark purple, and cytoplasm as light pink. In Fig. 5(b), color remapping process was described for each main structure, i.e., collagen and nuclei. The L channels for those structures were inverted to reflect the color intensity inversion, so the bright purple nuclei in the original UV-fluor data appear dark just as shown in MT staining. As for collagen, all of the channels were flipped to reflect not just the inversion in intensity, but also the hues to truly reflect the color transition in MT staining. The histograms of each channel (L, a, and b) in the L*a*b color space show that the distribution of intensity and hue information was preserved after the remapping process.

### 3.3 Classification of Stroma in PDAC Tumors is Feasible in Fresh Tissue via UV-fluorescence Imaging

**Fig. 6:**
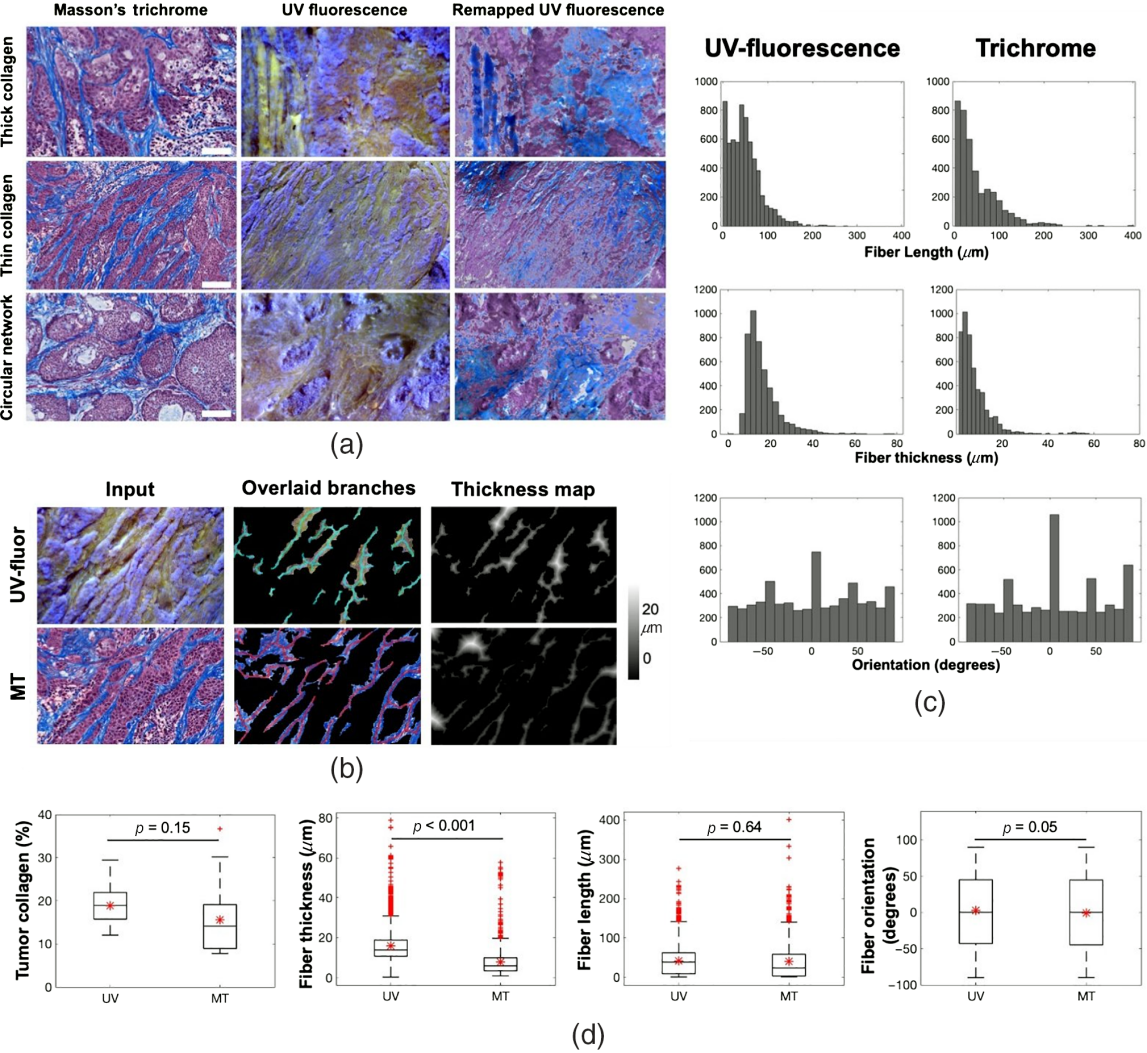
Quantification of collagen content in PDAC tumors. (a) Different types of collagen observed in both trichrome and UV-fluor data sets. (b) Quantification of collagen fibers. (c) Distribution of fiber length, fiber thickness, and fiber orientation obtained from both data sets (d) Statistical analysis was performed to show that there is no significant difference in means in terms of tumor collagen content, fiber length, and fiber orientation between UV-fluor and trichrome data. However, there was a statistical difference between two data sets for fiber thickness. Mean and standard deviation of UV versus trichrome data, respectively, for each category are 18.8±4.2 versus 15.6±8.1 (collagen content %), 15.9±7.8 versus 7.9±7.3 (collagen thickness in μm), 48.5±36 versus 49.3±47 (collagen fiber length in μm), 2.53±51 versus −0.85±51.3 (collagen fiber orientation in degrees). Data were analyzed for five tumors, necrosis areas were excluded, 50 ROIs were randomly selected with ROI size of 0.6×0.4  mm each, total collagen strands from UV data=6442, from trichrome data=6606.

Heterogeneity of collagen formation in PDAC tumors was captured and classified in Fig. 6. Collagen fiber visualization general agreement between UV-fluor and trichrome data was visualized in Fig. 6(a). Exact matching between two data sets was not expected due to tissue deformation and tissue changes due to pathology staining process to create trichrome-stained samples. However, direct comparison of the same tissue regions confirms the capability of UV-fluor imaging to visualize collagen in *ex vivo* samples. Quantification of the fluorescence signals also shows a strong agreement with results obtained from trichrome data. Figure 6(b) shows the outputs of the fiber strand analysis based on fiber thickness maps for both data sets.

The same algorithm was showed to work well for both. The distribution of collagen fibers in terms of length, thickness, and orientation was shown in Fig. 6(C) for both data sets to validate the feasibility of extracting quantitative information from UV-fluor imaging on fresh tissues. Results from statistical analysis in Fig. 6(d) shows that there was not a significant difference in means between the two data sets in terms of collagen content percentage, collagen fiber length, and orientation. About 20% difference in means ($\mu_{\mathrm{UV}}=18.8\%$ versus $\mu_{\mathrm{MT}}=15.6\%$) reported from comparing collagen content data was largely due to the differences in fiber thickness analysis ($\mu_{\mathrm{UV}}=15.9\;\mu m$ versus $\mu_{\mathrm{MT}}=7.9\;\mu m$), which could be largely attributed to the difficulty of accurate segmentation and separation of fibers from imaging bulk tissue. Another reason for thickness inconsistency was the thresholding applied on the collagen thickness map mentioned in Sec. 2. Better image quality with minimized out-of-focus pixels would significantly minimize the inaccuracy from thresholding and close the gap of fiber thickness reported by these two imaging modalities. Total count of collagen strands from both data sets is <3% different (6442 strands from UV data and 6606 strands from trichrome data). Fiber length on average is accurately reported with a discrepancy of less than 2% ($\mu_{\mathrm{UV}}=48.5\;\mu m$ versus $\mu_{\mathrm{MT}}=49.3\;\mu m$) while orientation shows a difference of 3.4 degrees on average.

### 3.4 UV-Fluorescence Imaging Could be Utilized as an Assay Platform to Evaluate the Effects of Photodynamic Priming on Collagen Modulation

**Fig. 7:**
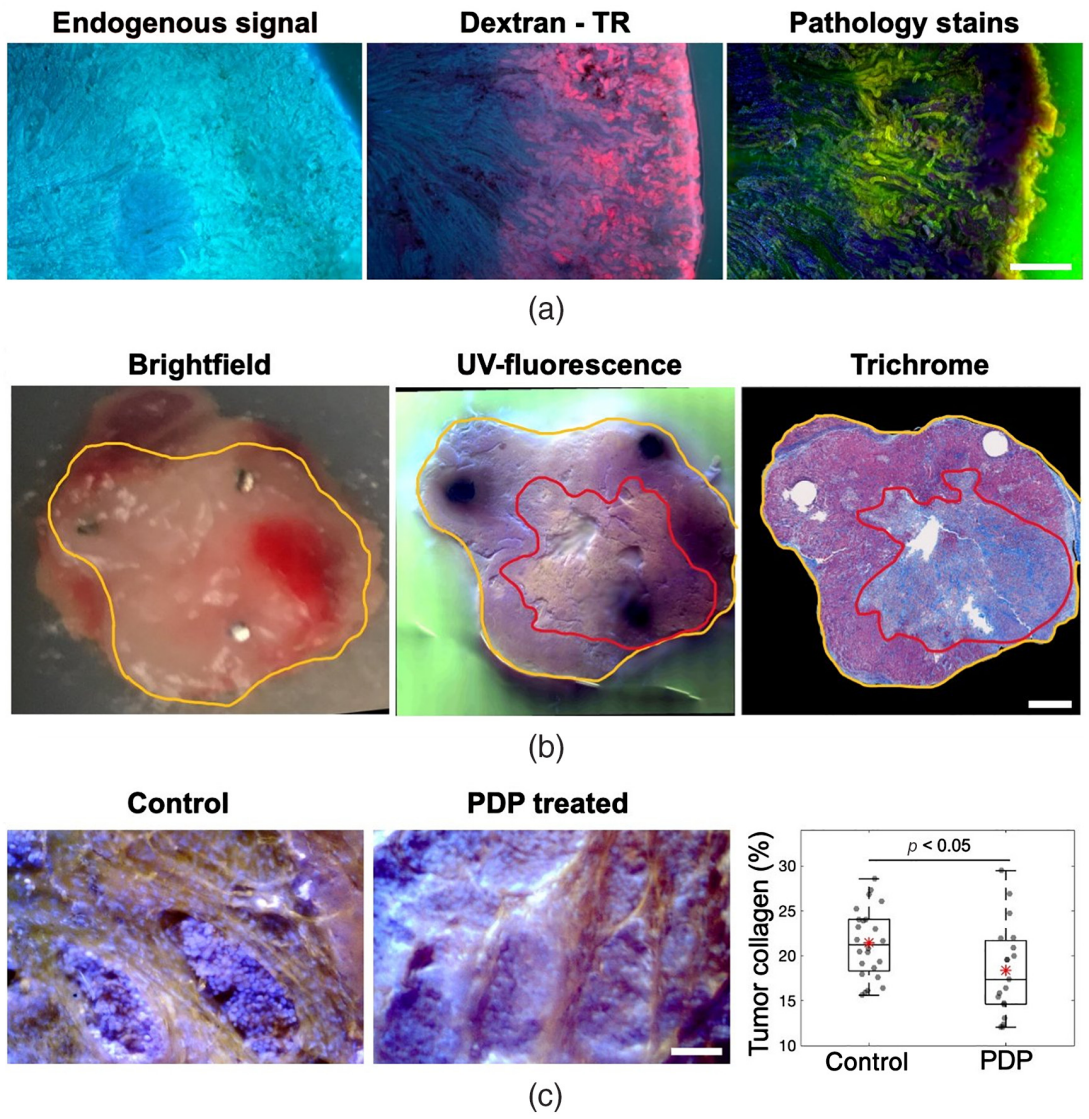
UV-fluor imaging as a fresh tissue assay platform to evaluate photodynamic priming responses. (a) Perfusion imaging of dextran tagged with TR in kidney samples. UV-excited TR shows perfused dextran in kidney samples (middle) as compared to endogenous fluorescence without any dextran injection (left). Dextran-perfused tissues could then undergo pathological staining (right) to obtain structural information, scale bar=500  μm. (b) Tissue imaging surface was outline in yellow. Necrosis outlined in red is hard to distinguish in brightfield image (left), but observable under UV-fluor imaging (middle), which is confirmed by trichome staining (right), scale bar=1  mm. (c) A comparison of collagen content in control and PDP treated tumors. Collagen modulation effect was observed, in which the PDP treated tumors show a 13% reduction in collagen content (n=2 animals per group, control ROIs=23, treated ROIs=19), scale bar=100  μm.

Figure 7 showcases additional features of UV-fluor imaging in fresh tissues that can be utilized in targeted therapy response assessments. Figure 7(a) shows the capability of imaging perfusion using the same simple imaging optics. Emission from TRexcited by 275-nm illumination was captured by the red channel on the RGB camera. Dextran-perfused samples could then be stained to obtain structural information without interference from TR signals. In Fig. 7(b), yellow outline was drawn to identify the tissue imaging surface while red outline locates the regions of necrosis due to photodynamic priming effect. This illustration highlights the capability of visualizing necrosis areas in fresh tissues, which normally is hard to delineate in brightfield images therefore in need of pathological confirmation. Furthermore, the capability of imaging collagen *in situ* demonstrated in this study could be utilized to assess the collagen modulation effect as an outcome of acute photodynamic priming, as reported in Obaid et al.^23^ Preliminary data acquired by UV-fluor imaging have showed possible reduction in desmoplasia for PDAC treated tumors in Fig. 7(c), which suggests that collagen content was reduced by 13%.

## 4 Discussion

This study was designed to test the feasibility of using UV-fluor imaging to extract quantitative morphological information from fresh PDAC tumors in xenograft models, at both microscopic and macroscopic levels. The combination of PDAC tumor’s desmoplastic nature, collagen-eosin fluorescence enhancement, and superficial optical sectioning of deep UV illumination have made stromal imaging in fresh tissue feasible. Results from Figs. 4 and 6 show the high fidelity of imaging PDAC stroma using this technique, with quantitative information comparable to MT staining data. Collagen fibers were found to be in the range of 5- to 30-μm thickness, with the majority of lengths to be under 50  μm and organized in a chaotic orientation. This is the first time that PDAC tumor stroma was visualized and quantified directly from fresh tissues under UV excitation light. Microscopy with UV surface excitation has been used on a variety of tissues and organs to demonstrate its capability of replacing H&E staining,^24^^,^^25^ however, stromal signals have not been a major focus. PDAC tumors have inherent desmoplasia, often consisting of thickened, heavily cross-linked collagen fibers. This abnormally high content of stroma and its direct, well-established relationship to progression and drug transport resistance makes this study particularly useful to assess the responsiveness of PDAC tumors to experimental therapies. Image data to showcase fresh collagen imaging from BxPC-3 tumors in this study suggests that future directions to study other pancreatic tumor types would be beneficial. It has been observed from other pancreatic tumor studies that the collagen fiber shape varies with different tumor types. One of our previous studies showed that more pronounced stiffness heterogeneity was linked to tumors with thicker collagen strands, which inversely affects drug perfusion.^17^

Studies since the 1960s have observed the enhancement of collagen fluorescence especially when introduced to eosin staining,^26^ and there are many speculations on the mechanism of why collagen signals are enhanced by eosin. Despite the lack of a clear mechanism, the fact that eosin is excited by deep UV and produces strong emission has been part of the realization that imaging collagen in fresh tissue would be possible without fixation. Our empirical results show that while rhodamine helped stain the cytoplasm, eosin enhanced the collagen fluorescence in PDAC tumors. This approach provides a very flexible and cost-effective tool for thin section imaging of surface-exposed tissues, instead of relying solely on collagen autofluorescence. Perhaps the most important factor in this is that this approach provides high-resolution images that can be achieved without fixation or thin section cutting, nor any post imaging registration of the images for analysis. This study also demonstrated color transfer with the goal of remapping UV-fluor to a conventional color scheme such as MT. While UV-fluor imaging intrinsically acquires data at a larger axial thickness as compared to MT, better image quality especially in image contrast will help improve fiber segmentation and collagen strand analysis so that errors in fiber thickness could be reduced. Future studies will involve improvements in these aspects.

The potential of imaging collagen using UV-fluor is even more prominent due to the capability of wide-field imaging to capture whole-specimen field of view. Due to the highly irregular tissue surface, collagen visualization with this technique does not provide the same image contrast as would be expected from other collagen imaging methods such as second harmonic imaging. However, the short exposure time (50 ms per frame) allows image acquisition of whole specimen within minutes. Wide-field imaging at microscopic resolution of tumor cells and collagen content offers an efficient tool for assessment of macroscopic targeted therapy responses, in terms of collagen modulation, tumor necrosis and tumor perfusion. Figure 7 shows additional features of this imaging tool besides stromal identification. Perfusion imaging was demonstrated by intravenously injecting dextran tagged with TR in the mice. Kidney samples in Fig. 7(a) showed TR emitted strongly when excited with 275-nm light source. That same tissue sample then could be stained as fresh tissues and/or fixed, sectioned, and stained to reveal structural information. The selective staining of nuclei using Hoechst allowed necrosis assessment as displayed in Fig. 7(b). With preliminary data in Fig. 7(c) showing possible reduction in desmoplasia due to acute PDP, a future application of this imaging technique aims to evaluate treatment responses of photodynamic priming on PDAC tumors, a targeted therapy that was proven to cause necrosis and modulate collagen,^23^ which resulted in better drug delivery.^20^

## 5 Summary

This study has demonstrated that high-resolution wide-field collagen imaging is feasible in fresh PDAC tumor tissues, with the employment of conventional pathology dyes and deep UV-illumination. Collagen quantification obtained from this UV-fluor imaging can be as quantitatively useful as data from MT stained thin sections but can be taken without the need for fixation, cutting, and post processing for registration. Additional features such as perfusion imaging and necrosis assessment make this simple imaging tool an attractive technique to evaluate targeted therapies such as PDT.

## Code, Data, and Materials Availability

MATLAB code used in this paper will be made available online.
